# The prognostic significance of BMI1 expression in invasive breast cancer is dependent on its molecular subtypes

**DOI:** 10.1007/s10549-020-05719-x

**Published:** 2020-06-10

**Authors:** Maryam Althobiti, Abir A. Muftah, Mohammed A. Aleskandarany, Chitra Joseph, Michael S. Toss, Andrew Green, Emad Rakha

**Affiliations:** 1 Division of Cancer and Stem Cells, Nottingham Breast Cancer Research Centre, University of Nottingham Biodiscovery Institute, University Park, Nottingham, NG7 2RD UK; 2 Department of Clinical Laboratory Science, College of Applied Medical Science, Shaqra University 33, Shaqra, 11961 Saudi Arabia; 3 Department of Pathology, Faculty of Medicine, Faculty of Medicine and Health Science, University of Benghazi, Benghazi, Libya; 4 Department of Histopathology, Nottingham University Hospital NHS Trust, City Hospital Campus, Hucknall Road, Nottingham, NG5 1PB UK

**Keywords:** BMI1, Breast cancer, Oestrogen receptor positive, Outcome, Breast cancer stem markers

## Abstract

**Purpose:**

BMI1, which is a major component of the polycomb group complex 1, is an essential epigenetic repressor of multiple regulatory genes and has been identified as a cancer stem cell (CSC) marker in several cancers. However, its role in breast cancer (BC) remains to be defined. In this study, we have evaluated the prognostic significance of BMI1 among the different molecular subtypes and assessed its association with other breast CSC markers (BCSC).

**Material and method:**

*BMI1* copy number and mRNA was assessed in large and well-characterised cohorts of early-stage BC patients [METABRIC (*n* = 1980) and the Bc-GenExMiner (*n* = 9616) databases]. BMI1 protein expression was assessed using tissue microarray and immunohistochemistry in a cohort of 870 invasive BC patients with long-term outcome data and the expression of a panel of BCSC markers was monitored.

**Result:**

BMI1 expression, prognostic significance and its association with BCSC markers were differed between molecular classes. In the luminal oestrogen receptor-positive (ER+) BC, BMI1 showed significantly higher expression compared to ER− tumours. BMI1 showed positive correlation with favourable prognostic features and it was negatively associated with the expression of key BCSC markers (ALDH1A1, CD24, CD44, CD133, SOX10 and SOX9). High expression of BMI1 was associated with longer breast cancer-specific survival (BCSS) independent of other prognostic variables. In the basal triple negative BC subtype, BMI1 expression showed positive association with CD133 and SOX10 and it was significantly associated with shorter BCSS.

**Conclusion:**

High BMI1 expression is associated with clinicopathological variables and outcome in BC. However, this association is dependent on the molecular subtypes. Further functional assessment to detect its underlying mechanistic roles in BC subtypes is warranted.

**Electronic supplementary material:**

The online version of this article (10.1007/s10549-020-05719-x) contains supplementary material, which is available to authorized users.

## Introduction

Polycomb complex protein or B-lymphoma Moloney murine leukaemia virus insertion region-1 *(*BMI1) is a member of the polycomb family which are a group of transcriptional repressors [1, 2]. BMI1 has a RING finger at the N-terminus, a central helix-turn-helix domain and a carboxyl-terminal PEST-like domain at the C-terminal end. The RING domain is required for BMI1 to localise to DNA strand breaks; therefore, it is involved in DNA damage response. The central helix-turn-helix domain with RING domain increases the life span of the cell. PEST domain is required for protein degradation [3]. BMI1 has been reported to be involved in several different pathways such as Wnt, Akt, Notch and Hedgehog signalling [4–7]. Hence, BMI1 has been shown to behave as a key regulator in the self-renewal, differentiation and tumour initiation of breast cancer stem cells (BCSC) [8]. In in vitro models, it has been observed that overexpression of BMI1 is linked to increased cell motility and invasion in BC [9, 10]. Arnes et al. have reported that low expression of BMI1 is associated with high expression of Aldehyde dehydrogenase 1 (ALDH1) in African BC patients, where ALDH1 has been used as a functional marker to define the BCSC [11]. BMI1 has also been considered as a poor prognostic and predictive biomarker in several types of cancer [1, 12, 13]. However, conflicting data have been reported in the same study where high expression of BMI1 at mRNA and protein levels was associated with high expression of ER and positive axillary lymph node metastasis, leading to some difficulty in the interpretation of the results [14].

There is little available evidence on the immunohistochemical expression of BMI1 in BC tissue samples and whether it is also considered a biomarker of poor or good prognosis in BC. This study aimed to investigate the clinical and pathological relevance of BMI1 expression, including its genomic, transcriptomic and protein levels, in BC utilising large cohorts of early-stage BC with a long-term follow-up. This is to characterise the variation of BMI1 expression in different BC molecular subtypes and to explore the associations between BMI1 and a panel of relevant BCSC markers at both the mRNA and protein levels.

## Material and methods

### BMI1 protein expression

The study cohort comprised 870 invasive BCs derived from the retrospective Nottingham Primary Breast Carcinoma Series of patients presenting to Nottingham City Hospital between 1986 and 1998. Patients’ clinical and pathological data including age at diagnosis, histological tumour type, tumour size, lymph node status, Nottingham Prognostic Index (NPI), lympho-vascular invasion (LVI) and adjuvant therapy were available and prospectively maintained. Data for oestrogen receptor (ER), progesterone receptor (PgR), HER2 status and Ki67 data were available [15, 16]. ER and PgR cut-off values were defined as ≥ 1% and HER2 status was defined as previously published [15, 17, 18]. Survival data were accessible and prospectively maintained including the following: (1) BC-specific survival (BCSS), defined as the time (in months) from the date of the primary surgical treatment to the time of death from breast cancer, and (2) distant metastasis free survival (DMFS), defined as the time (in months) from the surgery until the first event of distant metastasis [19]. The clinicopathological parameters for the study cohort are summarised in supplementary Table 1. BC intrinsic molecular subtypes were determined as previously descried [20]. Immunohistochemical detection of a panel of BCSC, including ALDH1A1, CD133, CD24, CD44, EPCAM, SOX9 and SOX10, had been previously performed [21–23] and these were used in the current study to assess their relationship with BMI1 expression.

### Tissue microarrays (TMAs) and immunohistochemical (IHC) evaluations

Full face BC tissue sections were stained using IHC to evaluate the pattern of immunohistochemical BMI1 expression prior to staining of TMAs. Kidney tissue was used as a positive control while the negative control was obtained by omitting the application of primary antibody in the IHC staining protocol.

Formalin-fixed paraffin-embedded BC tissue samples were arrayed as previously described [24]. Prior to IHC staining, the specificity of the anti-BMI1 antibody was validated using Western blotting in MCF7, MDA-MB-231, SKBR3, MDA-MB-468 BC cell lines’ lysates (American type culture collection, Rockville, MD, USA) and HeLa cells as control. This was performed using 1:5000 dilution of the primary antibody (EPR3745 (2), Abcam, UK), and 1:15,000 of the horseradish peroxidase-labelled secondary anti-rabbit antibody, with b-actin (1:5000) used as a loading control. A single band for BMI1 was observed at the predicted size (40 kDa), which confirmed the specificity of the antibody (Supplementary Fig. 1). IHC staining was performed on 4 μm TMA sections using Novolink polymer detection system (Leica, Newcastle, UK). In brief, the antigen retrieval was performed in citrate buffer (pH 6) in a microwave (Whirlpool JT359 Jet Chef 1000 W) for 20 min. The optimal dilution of BMI1 antibody in IHC was 1:100 and incubated for 1 h at room temperature. Stained TMA slides were scanned with high-resolution digital images (NanoZoomer; Hamamatsu Photonics, Welwyn Garden City, UK), at 20 × magnification and viewed by Xplore viewer (Philips, Belfast UK). The BMI1 staining TMA cores were evaluated on the basis of a semiquantitative scoring of core digital images using a modified histochemical score (*H*-score) [25]. All cases were scored by M. Althobiti, blinded to histopathological data and patients’ outcome. To validate the results and test for the inter-observers reproducibility of the scoring, 10% of the cases were randomly selected and rescored by another observer (M. Toss).

### Genomic and transcriptomic analysis

A cohort of 1980 BC patients was evaluated in terms of *BMI1* gene copy number (CN) aberrations and mRNA expression using the Molecular Taxonomy of Breast Cancer International Consortium (METABRIC) [26, 27]. The cut-off point of *BMI1* was determined using X-tile software (version 3.6.1, Yale University, USA), which was based on prediction of BCSS. The clinicopathological parameters of METABRIC series are summarised in supplementary Table 1. There was no difference in the distribution of clinicopathological parameters between Nottingham series and METABRIC series of patients (correlation coefficients = 0.733, all *P* < 0.0001) [28].

**Table 1:** The association of BMI1 and clinicopathological parameters in breast cancer in (Protein and mRNA levels)

Parameters	Protein expression (*n* = 870)			mRNA expression (1980)		
	Low BMI1 No (%)	High BMI1 No (%)	x^2^ *P* value	Low BMI1 No. (%)	High BMI1 No. (%)	x^2^ *P* value
Patient age (years)						
< 50	451 (80)	111 (20)	0.22	147 (37)	249 (63)	11.809
≥ 50	243 (79)	65 (21)	0.635	411 (28)	1047 (72)	**0.001**
Tumour size (cm)						
< 2	369 (82)	83 (18)	1.77	193 (31)	429 (69)	4.218
≥ 2	319 (78)	90 (22)	0.106	476 (36)	855 (64)	0.040
Tumour grade						
Grade I	90 (69)	40 (31)		20 (12)	143 (88)	
Grade II	211 (75)	69 (25)	22.33	162 (22)	567 (78)	91.336
Grade III	385 (86)	64 (14)	** < 0.0001**	357 (41)	523 (59)	** < 0.0001**
Tubules formation						
Score 1	26 (67)	13 (33)				
Score 2	219 (76)	70 (24)	11.02	N\A		
Score 3	420 (83)	84 (17)	**0.004**			
Mitotic count						
Score 1	187 (73)	68 (27)				
Score 2	131 (77)	39 (23)	14.99	N\A		
Score 3	347 (85)	60 (15)	** < 0.0001**			
Nuclear pleomorphism						
Score 1	11 (78.6)	3 (21)				
Score 2	223 (72.2)	86 (28)	18.76			
Score 3	431 (84.7)	78 (15)	** < 0.0001**	N\A		
Axillary nodal stage						
Stage I	426 (80)	103 (20)	0.65	281(29)	683(71)	
Stage II	214 (79)	57 (21)	0.721	29.4(29)	414(71)	3.418
Stage III	46 (77)	14 (23)		34.5(34)	199(66)	0.181
Nottingham prognostic index						
Poor prognostic group	119(83.8)	23(16.2)		135(21)	508(79)	
Moderate prognostic group	389(81.7)	87(18.3)	7.46	346(34)	676(66)	42.356
Good prognostic group	180 (74.1)	63(25.9)	**0.024**	77(41)	112(59)	** < 0.0001**
Oestrogen receptor						
Negative	213(91)	20(9)	26.12	224(56)	177(44)	165.226
Positive	474(76)	152(24)	** < 0.0001**	319(23)	1095(77)	** < 0.0001**
Progesterone receptor						
Negative	309(87)	48(13)	14.48	368(42)	502(58)	116.00
Positive	361(76)	114(24)	** < 0.0001**	190(19)	794(81)	** < 0.0001**
HER2 status						
Negative	557 (79)	151 (21)	8.46	467 (29)	1157 (71)	10.616
Positive	103 (90)	11 (10)	**0.002**	90 (39)	139 (6)	**0.001**

Bold represents the significant *P* values

To validate the prognostic significance of *BMI1* mRNA expression, another publically available database (Breast Cancer Gene Expression Miner v4.0 (Bc-GenExMiner v4.0), with the online dataset available at https://bcgenex.centregauducheau.fr), was used. This large dataset (*n* = 9616) allowed the evaluation of the prognostic role of *BMI1* in BC cohorts, which include key prognostic parameters such as patients’ age, tumour grade, nodal status, NPI, ER and molecular subtypes. Univariate analyses for molecular BC subtypes were performed [29].

### Statistical analysis

IBM SPSS 24.0 (Chicago, IL, USA) software was used for statistical analysis. The inter-observer agreement was determined using intra-class correlation coefficient. BMI1 expression was categorised using 9.1 and 130 H-score cut-off of transcriptomic and IHC expression, respectively. Both cut-offs were determined using x-tile Bioinformatics software version 3.6.1 (Yale University, USA). The association between the categorical groups of BMI1 and clinicopathological parameters was analysed using a Chi-square test. The correlation of BMI1 and other biomarkers was tested using continuous data and Spearman test for the IHC analysis while the person test used for mRNA expression data. Associations with patient outcome were assessed using the Kaplan–Meier survival curves and the log-rank test. Cox proportional hazards regression models were built for multivariate survival analyses to estimate the hazard ratio (HR) of BMI1 adjusted by other well-known prognostic factors. A *P* value of less than 0.05 (two- tailed) was considered significant in all statistical tests.

## Results

### BMI1 expression in BC

**Fig. 1:**
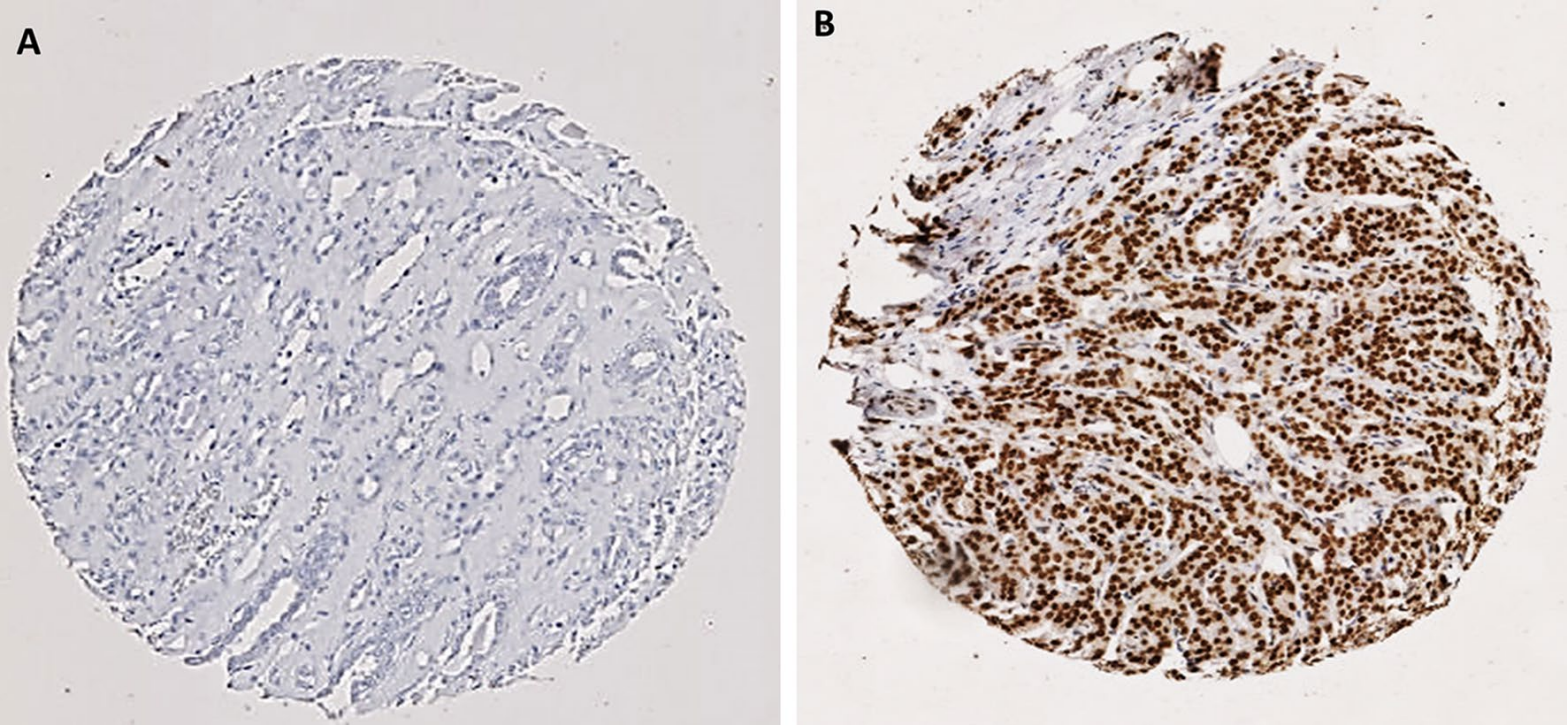
Representative photomicrographs of the expression of BMI1 in invasive breast cancer **a** negative immunohistochemical (IHC) expression. **b** Positive IHC expression

**Fig. 2:**
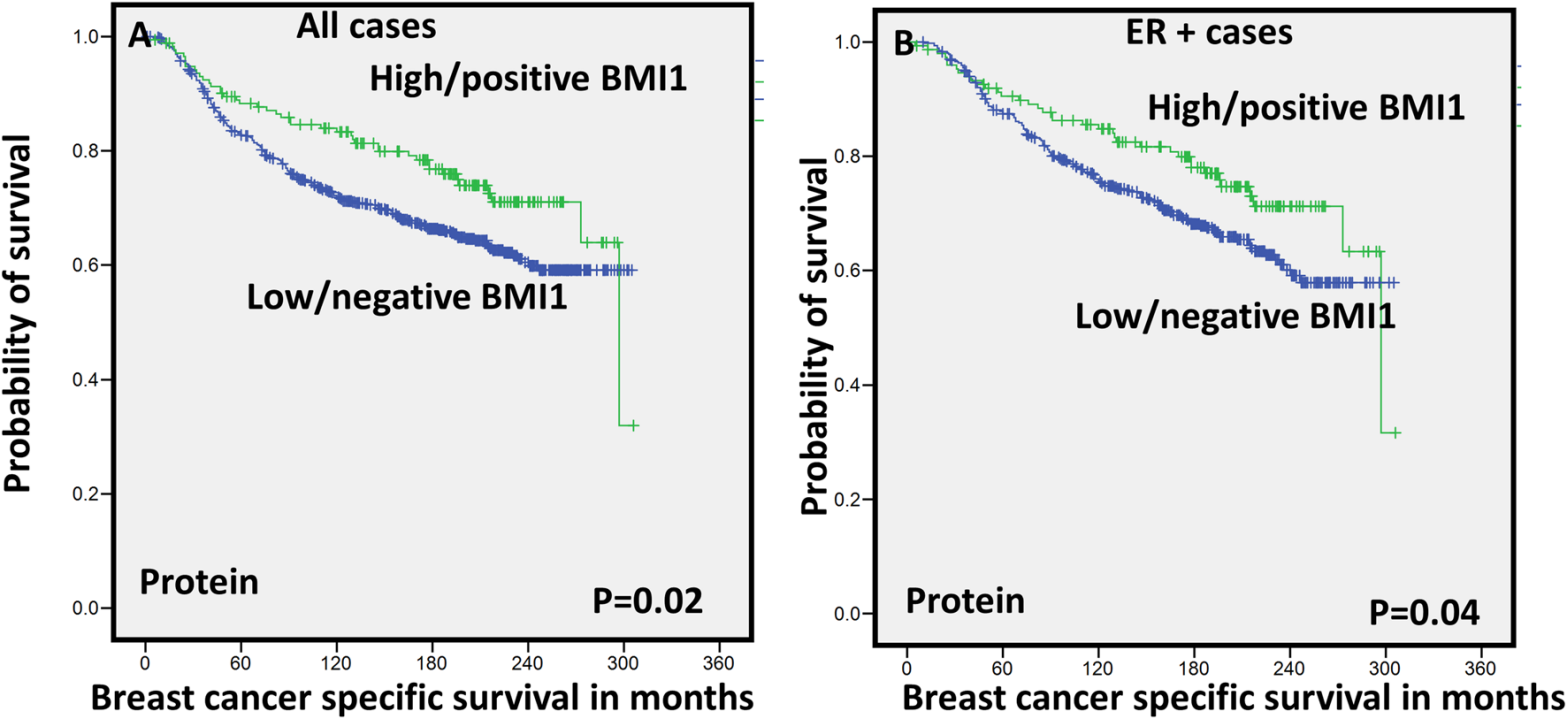
Kaplan Meier survival plots for BMI1 expression (Protein) **a** breast cancer-specific survival (BCSS) in all cases and **b** breast cancer-specific survival (BCSS) in ER + cases

The IHC staining showed a homogenous staining pattern, with BMI1 expression localised in the nuclei of the invasive tumour cells. The staining intensities varied from negative (no stain) to strong intensity (Figs. 1, 2). Inter-observer agreement was determined, and the interclass correlation coefficient was 0.931, indicating an excellent concordance between the 2 scorers.

In the whole BC cohort, the IHC expression of BMI1 ranged from 0 to 270 H-score. The data showed that high/positive BMI1 IHC expression (H-score > 130) was observed in 20% of cases (176/870), whereas 80% of cases (694/870) was considered low/negative. Interestingly, the immunoexpression of BMI1 in the luminal ER-positive (ER+) subtype was higher than in ER-negative (ER−) subtypes (*P* < 0.0001). In the METABRIC cohort, high expression of *BMI1* mRNA was seen in 65% of cases (1288/1980). In the Nottingham cases of the METABRIC cohort (*n* = 340), there was a strong association between *BMI1* mRNA expression and protein expression (*P* = 0.001)*. BMI1* CN gain was observed in 6% of cases (113/1980) whereas 1% of cases (25/1980) showed CN loss. Supplementary Table 2 summarises the mean, median and the range of expression of BMI1 in BC subtypes at both protein and mRNA levels.

**Table 2:** Multivariate Cox regression hazard model including other prognostic clinicopathological parameters shows that high BMI1 (immunohistochemically) provided an independent prognostic value, associated with longer breast cancer-specific survival in the whole cohort

Variable	Hazard ratio	95% Confidence interval (CI)		*p* value
		Lower	Upper	
Patient age	0.88	0.69	1.135	*0.346*
Tumour Grade	1.61	1.32	1.96	** < 0.0001**
Node stage	1.99	1.67	2.38	** < 0.0001**
Tumour size	0.67	0.52	0.87	**0.002**
Bmi1	0.72	0.51	0.99	**0.048**

Bold represents the significant *P* values

In this cohort, 73% of ER+ subtype expressed high *BMI1* while 60% of the ER-negative subtype expressed high *BMI1.*

### BMI1 and clinicopathological features

High BMI1 protein expression showed an association with clinicopathological parameters characteristic of good prognosis including lower histological grade (*P* < 0.0001), more tubule formation (*P* = 0.004), lower mitotic count (*P* < 0.0001), lower nuclear pleomorphism (*P* < 0.0001), lower NPI scores (*P* = 0.024), special tumour type of good prognosis (*P* < 0.0001) and with tumours showing ER+ (*P* < 0.0001) and HER2− phenotypes (*P* = 0.002) (Table 1).

Similar findings were identified in the METABRIC cohort. High expression of *BMI1* mRNA was positively associated with good prognostic factors, such as older age (*P* < 0.001), postmenopausal status (*P* < 0.001), lower grade (*P* < 0.0001), good NPI prognostic group (*P* < 0.0001), tumours of tubular subtype (*P* < 0.0001) and HER2− phenotypes (*P* = 0.001) as shown in Table 1.

These associations with good prognostic factors were also obtained in the BC Gene Expression Miner database; high expression of *BMI1* was associated with older age, good prognostic factors, such as good NPI and luminal A subtype (*P* < 0.0001), as shown in supplementary Fig. 2.

### Outcome analysis

Univariate survival analysis showed that high BMI1 protein expression was significantly associated with longer survival in terms of breast cancer-specific survival (BCSS) in the whole BC cohort (*P* = 0.02) (Fig. 2a). With regard to molecular subtypes, in the luminal ER+ tumours, high expression of BMI1 was significantly associated with BCSS (*P* = 0.04; Fig. 2b) and distant metastasis free survival (DMFS) (*P* = 0.04; supplementary Fig. 3). However, in the basal / triple negative subtype (TNBC), high expression of BMI1 was significantly associated with shorter BCSS (*P* = 0.04) (supplementary Fig. 3). In HER2-positive (HER2+) tumours, no significant association between BMI1 and outcome was identified.

**Fig. 3:**
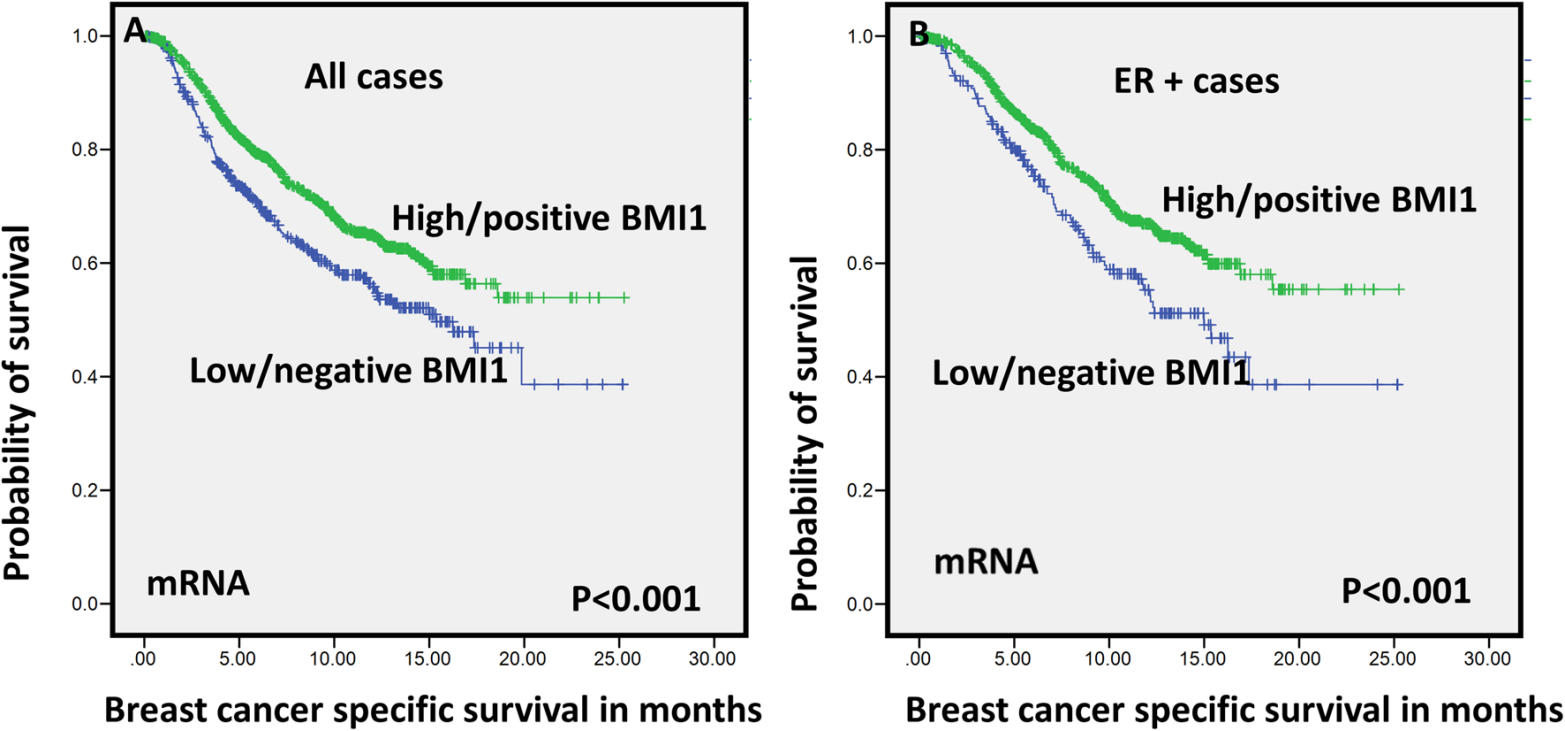
Kaplan–Meier survival plots for BMI1 expression (mRNA) **a** breast cancer-specific survival (BCSS) in all cases and **b** breast cancer-specific survival (BCSS) in ER + cases

The Cox regression model, including age at diagnosis, tumour size, tumour grade and nodal stage, showed that BMI1 as an independent predictor of good prognosis in the whole BC cohort and in the luminal ER+ patients (*P* = 0.04, HR 0.72; 95% Cl 0.51–0.99, *P* = 0.04, and HR 0.69; 95% Cl 0.48–0.99, respectively) (Tables 2, 3) but not in the TNBC subtype. When the multivariate analysis including BMI1 and other BCSC (ALDH1A1, CD133, CD24, and SOX9) markers, BMI1 was an independent predictor of good prognosis in the whole BC cohort (*P* = 0.017), supplementary Table 3.

**Table 3:** Multivariate Cox regression hazard model including other prognostic clinicopathological parameters shows that high BMI1 (immunohistochemically) provided an independent prognostic value, associated with longer breast cancer-specific survival in the luminal oestrogen receptor-positive breast cancer

Variable	Hazard ratio	95% Confidence interval (CI)		*p* value
		Lower	Upper	
Patient age	1.03	0.76	1.39	*0.824*
Tumour Grade	1.70	1.36	2.13	** < 0.0001**
Node stage	2.03	1.62	2.56	** < 0.0001**
Tumour size	0.63	0.46	0.86	**0.004**
Bmi1	0.69	0.48	0.99	**0.047**

Bold represents the significant *P* values

With regard to the transcriptomic expression, high *BMI1* mRNA expression in the METABRIC cohort was significantly associated with longer BCSS in the whole BC cohort (*P* < 0.001) as well as in the luminal ER+ tumours (*P* < 0.001) (see Fig. 3). However, no significant association between *BMI1* and outcome was observed in ER− tumours or in HER2+ patients (*P* > 0.05). Moreover, CN gain of *BMI1* was associated with longer BCSS (*P* = 0.031) in whole BC cohort.

Similar results were observed with the BC Gene Expression Miner dataset, where *BMI1* mRNA was associated with longer survival in the ER+ tumour (*P* = 0.020), which confirms the previous findings in the METABRIC cohort as shown in Supplementary Fig. 4.

### BMI1 and other biomarkers

We further investigated the association of BMI1 expression and key BC stem cell (BCSC) biomarkers. At protein level, there was a significant negative correlation between the high expression of BMI1 and BCSC markers: ALDH1A1 (*P* = 0.017), CD133 (*P* = 0.006) and SOX9 (*P* = 0.004) in all BC cases (supplementary Table 4a). In the ER+ tumours, BMI1 maintained similar associations and showed negative correlation with ALDH1A1 (*P* = 0.005) and CD133 (*P* = 0.001). Interestingly, in the ER- tumours, BMI1 showed positive association with CD133 (*P* < 0.0001) and SOX10 (*P* = 0.01) expression (supplementary Table 4a).

Similarly at the mRNA level, high expression of *BMI1 was* negatively associated with BCSC including *ALDH1A3* (*P* < 0.0001), *CD133* (*P* = 0.0003), *CD24* (*P* = 0.002) and CD44 (*P* = 0.0007) (Supplementary Table 4b) in the whole BC cohort. When the analysis was restricted to the luminal ER+ tumours, high *BMI1* expression was associated with low expression of *ALDH1A1* (*P* = 0.025), *ALDH1A3* (*P* = 0.026), *CD133* (*P* = 0.038), *CD44* (*P* = 0.0001), *CD24* (*P* = 0.004) and *SOX10* (0.0006) (Supplementary Table 4b). In the basal TNBC subtype, positive association was observed between *BMI1* and *EPCAM* and *SOX10* (*P* < 0.001).

## Discussion

BC is a heterogeneous disease and the understanding of this heterogeneity is of great importance to improve patient outcome and treatment regimens [30]. Studies have addressed and investigated prognostic factors or biomarkers that illustrate the complex clinical and biological differences in BC subtypes [28, 31, 32]. In the current study, BMI1 expression was evaluated at protein, transcriptomic and genomic levels in well-characterised cohorts of early-stage BC with different molecular subtypes of BC tissue samples.

In the current study, immunopositivity and mRNA of BMI1 were observed in 20% and 70% of BC. Previous studies have reported high BMI1 expression in 62% and 53% of BC [14, 33]. The difference in the frequency of positivity between our results and previous studies could be explained by the difference in the scoring method and varying the definitions of the positivity cut-offs. Our cohort was scored using H-scoring, which is a widely accepted system in both clinical and research settings and the previous studies used other scoring system [17, 34]. BMI1 has been linked with poor prognosis and shorter survival in several cancer types [35, 36]. However, its prognostic role in BC remains controversial. Engelsen et al. reported that low expression of BMI1 is associated with loss of ER and PR in endometrial carcinoma [37]. In BC, Kim et al. have reported that BMI1 is associated with positive lymph node metastasis but it was also associated with ER + tumours, which are less aggressive tumour compared with other subtypes of BC [14]. Choi et al. demonstrated that BMI1 is favourable prognostic biomarker in BC [33].

In this study, a significant correlation between high expression of BMI1 and good prognostic BC features was found in the whole cohort and in the ER+ tumours. In ER+ subtype, BMI1 was highly expressed compared to ER− classes. This is in an agreement with Choi et al. study, which showed the same findings and reported that IHC overexpression of BMI1 was associated with ER+ expression and other favourable clinicopathological parameters including smaller tumour size, negative lymph node metastasis and intermediate nuclear grade [33].

BMI1 was also here demonstrated to be an independent good prognostic biomarker in ER+ tumours, independently of other clinical pathological features [33]. Engelsen et al. have suggested a potential link between BMI1 and hormone receptor status [37]. Interestingly, although there was a limited case number of TNBC subtypes in that study cohort (*n* = 56), increased expression of BMI1 was associated with shorter survival in TNBC [38]. This finding suggests a diverse role for BMI1 in different BC subtypes.

BMI1 plays a vital function in the epigenetic regulation of stem cell transcriptional pathways and is also implicated in the self-renewal, proliferation and cell cycle of CSCs [39, 40]. To the best of our knowledge, this is the first study analysing the association of BMI1 with BCSC biomarkers at both transcriptomic and protein levels. Our data have shown that BMI1 is negatively correlated with some BCSC markers at both mRNA and protein levels in all BC and the ER+ subtypes; however, the association between BMI1 and BCSC markers in TNBC was limited (only CD133 and SOX10), which further supports a different role of BMI1 in relation to the molecular subtypes of BC particularly ER+ . Our data favour a BCSC function of BMI1 in TNBC but not in ER+ tumours, though this warrants further experimental validation. Our results also showed that the interaction between BMI1 and HER2 is limited and that BMI1 is not associated with outcome in HER2+ tumours.

## Conclusion

The current study indicates that BMI1 exhibited a varied role in BC subtypes. In ER+ tumours, BMI1 is associated with good prognosis and longer survival; however, in TNBC, BMI1 showed an association with shorter survival and shows different associations with BCSC markers, which suggest the ER status that may play an important role in modulating the biological function of BMI1. Further functional studies are essential to be performed in order to clarify the significant role of BMI1 in different BC subtypes.

## Electronic supplementary material

Below is the link to the electronic supplementary material.

Supplementary file1 (DOCX 412 kb)

Supplementary file2 (DOCX 30 kb)

## Data Availability

The dataset analysed during the current study is available from the corresponding author on reasonable request.
