# Supplementary material for: The prognostic significance of BMI1 expression in invasive breast cancer is dependent on its molecular subtypes
Source: Breast Cancer Res Treat. 2020 Jun 10;182(3):581–9. doi: 10.1007/s10549-020-05719-x (PMC7320923; doi:10.1007/s10549-020-05719-x)
Supplement: Supplementary file 1 — Supplementary file1 (DOCX 412 kb) [file 10549_2020_5719_MOESM1_ESM.docx]

**Supplementary fig1** Western blot of rabbit monoclonal anti-BMI-1 antibody showing a single specific band in (left green band) at expected molecular weight (40 kDa) in lanes 1) MCF7, 2) MDA-MB-231, 3) SKBR3, and 4) MDA-MB-468 Cell 5) Hela Lysates. The red bands in lanes 1, 2, and 3 represent the beta-actin (positive control) at 42 kDa molecular weight in 1) MCF7, 2) MDA-MB-231, 3) Hela cell lines, respectively.


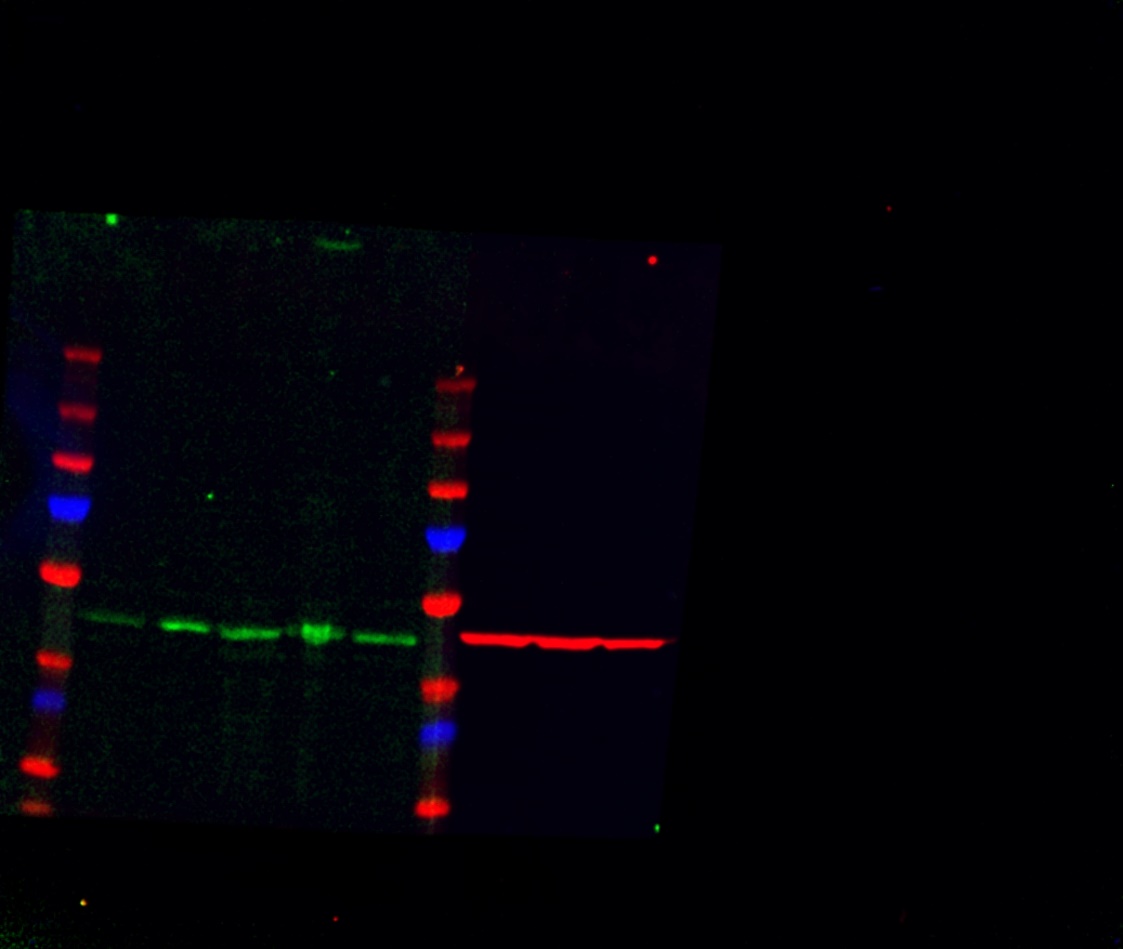


**1 2 3 4 5 1 2 3**

**B-actin**

**BMI1**

**Supplementary fig 2** *BMI1* mRNA expression and clinico-pathological parameters using BC gene-expression Miner in unselected cases.


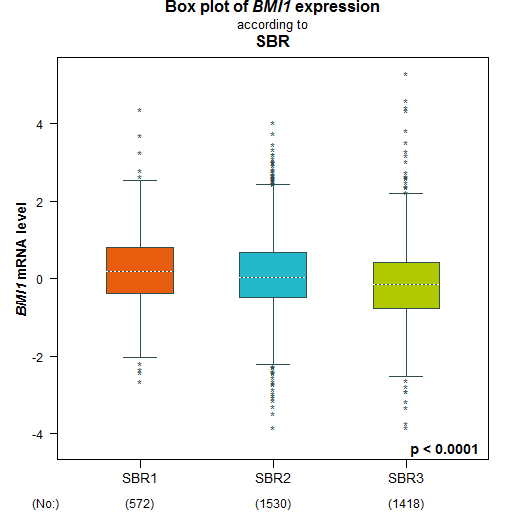

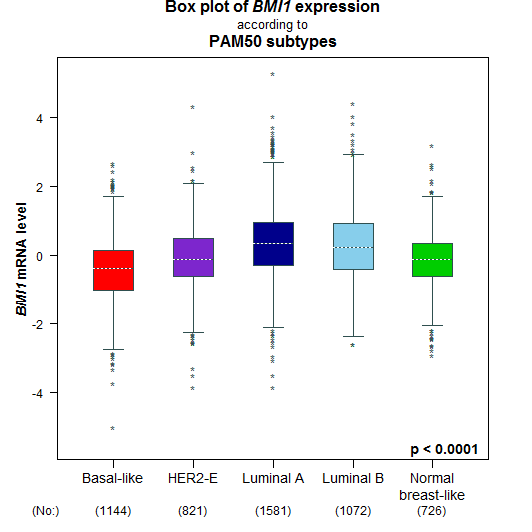

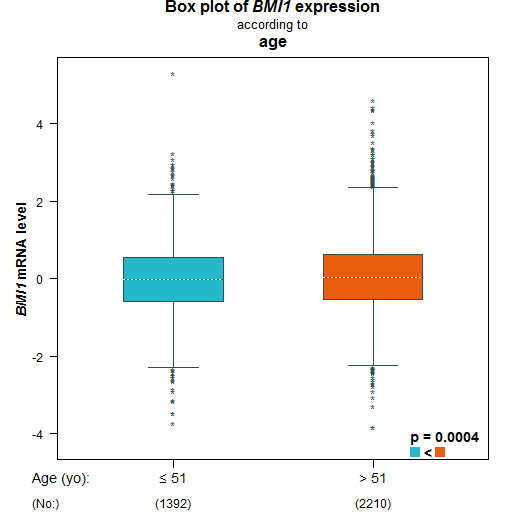


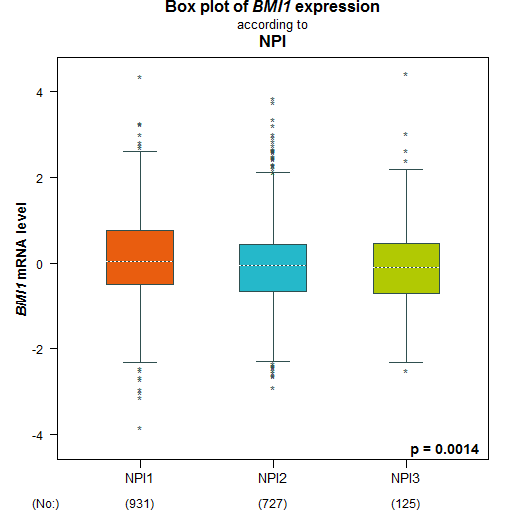


**Supplementary fig 3** Kaplan Meier survival plots for BMI-1 expression (Protein) in breast cancer for A) Distant Metastasis Free Survival (DMFS), B) breast cancer specific survival (BCSS) in Triple negative BC cases.


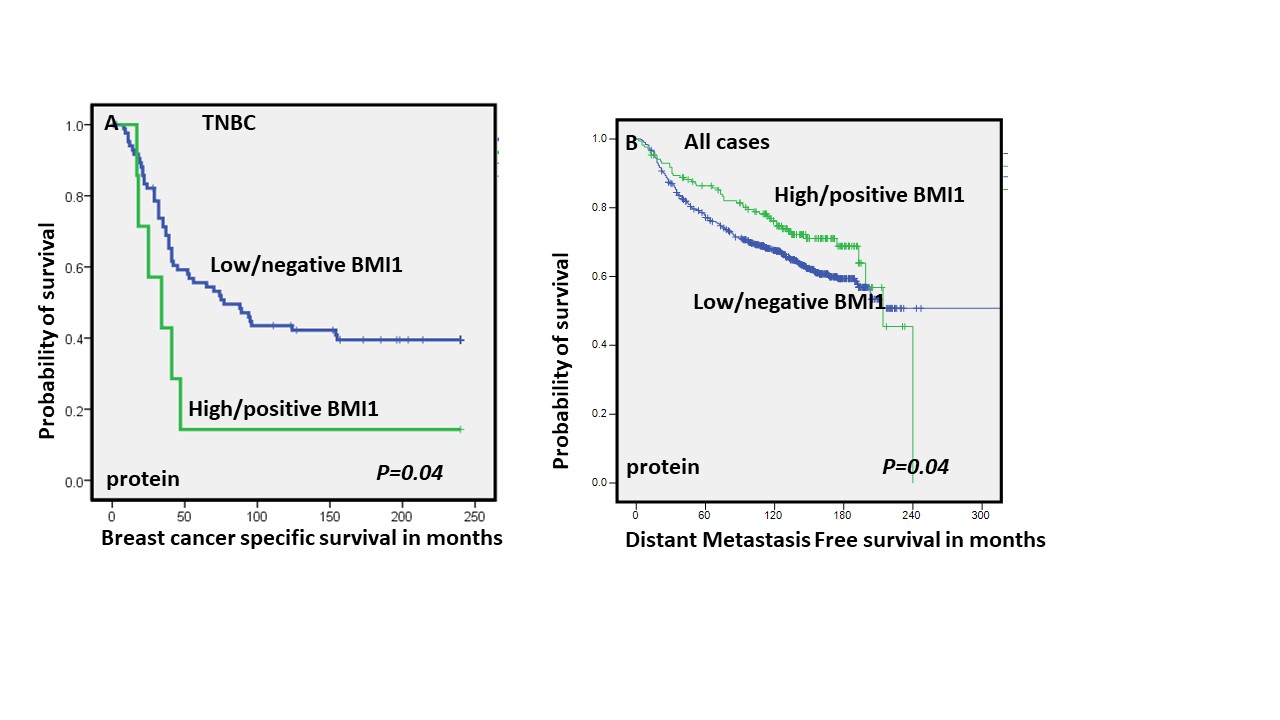


**Supplementary figures 4)** *BMI1* mRNA expression and BC patient outcome using BC gene-expression Miner in unselected cases (a), and ER+ subtype (B)

**A**


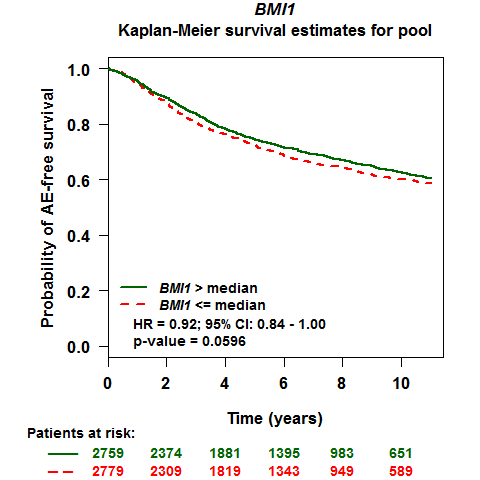

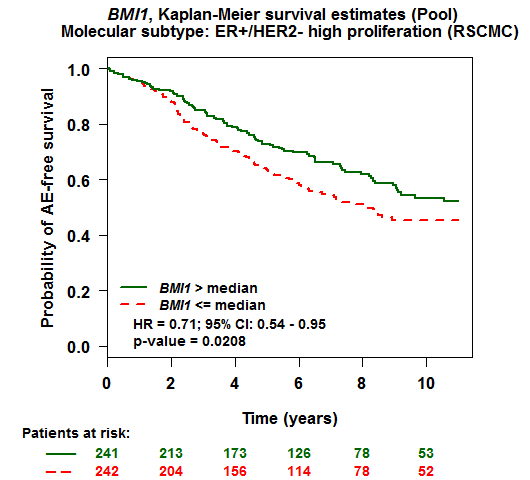


**B**
