# Supplementary material for: The prognostic significance of BMI1 expression in invasive breast cancer is dependent on its molecular subtypes
Source: Breast Cancer Res Treat. 2020 Jun 10;182(3):581–9. doi: 10.1007/s10549-020-05719-x (PMC7320923; doi:10.1007/s10549-020-05719-x)
Supplement: Supplementary file 2 — Supplementary file2 (DOCX 30 kb) [file 10549_2020_5719_MOESM2_ESM.docx]

**Supplementary table 1:** Summary of patient demographics of the cohorts used in this study

| **Parameters** | **Nottingham series**  **N (%)** | **METABRIC series**  **N (%)** |
| --- | --- | --- |
| **Patient Age (Years)**  < 50  ≥ 50 | 562(65)  308(35) | 396(21)  1458(79) |
| **Tumour size (cm)**  ≤ 2  > 2 | 409(41)  452(59) | 622(32)  1331(68) |
| **Tumour Grade**  Grade I  Grade II  Grade III | 130(15)  280(33)  449(52) | 163(9)  729(41)  880(50) |
| **Axillary nodal stage**  Stage I  Stage II  Stage III | 529(62)  271(32)  60(6) | 964(59)  443(27)  234(14) |
| **Nottingham Prognostic Index**  Poor Prognostic Group  Moderate Prognostic Group  Good Prognostic Group | 142(17)  476(55)  243(28) | 643(35)  1022(55)  189(10) |
| **Oestrogen Receptor**  Negative  Positive | 233(27)  626(73) | 401(22)  1414(78) |
| **Progesterone Receptor**  Negative  Positive | 357(43)  475(57) | 870(47)  984(53) |
| **HER2 status**  Negative  Positive | 708(86)  114(14) | 1624(88)  229(12) |
| **Follow-up status**  Alive  Died from BC | 440(61)  280(39) | 1071(68)  505(32) |

**Supplementary table 2:** Mean, median and range of percentages of BMI-1 expression in the whole cohort and different breast cancer molecular subtypes

| **BMI-1 Expression** | **Immunohistochemical expression** | | | **mRNA expression** | | |
| --- | --- | --- | --- | --- | --- | --- |
|  | **Whole cohort** | **Luminal ER+ BC subtype** | **TNBC subtype** | **Whole cohort** | **Luminal ER+ BC subtype** | **Basal ER- BC**  **subtype** |
| Mean | 81.73 | 93.91 | 47.80 | 9.5 | 9.6 | 9.1 |
| Median | 80 | 90 | 30.00 | 9.4 | 9.5 | 9 |
| Range | 0-270 | 0-270 | 0-240 | 7.6-12 | 7.8-12 | 7.6-11.5 |

**Supplementary table 3:** Multivariate Cox regression hazard model including other breast cancer stem cell (BCSC) markers shows that high expression of BMI-1 (immunohistochemically) provided an independent prognostic value; associated with longer breast cancer specific survival in whole BC cohort.

| **Variable** | **Hazard ratio** | **95% Confidence Interval (CI)** | | **P-value** |
| --- | --- | --- | --- | --- |
|  |  | Lower | Upper |  |
| ALDH1A1 | 0.999 | 0.995 | 1.002 | 0.468 |
| CD133 | 0.999 | 0.994 | 1.003 | 0.504 |
| CD24 | 1.001 | 0.999 | 1.003 | 0.166 |
| SOX9 | 1.007 | 1.002 | 1.012 | **0.008** |
| BMI-1 | 0.996 | 0.992 | 0.999 | **0.017** |

| **BCSC markers** | **BMI-1 expression (Whole cohort)**  Correlation coefficient (p-value) | **BMI-1 expression (Luminal ER+BC)**  Correlation coefficient (p-value) |
| --- | --- | --- |
| **ALDH1A1** | -0.101 **(0.017)** | -0.150 (**0.005**) |
| **CD133** | -0.123 **(0.006)** | -0.171 (**0.001**) |
| **CD44** | -0.046 (0.321) | -0.004 (0.766) |
| **CD24** | -0.090 **(0.023)** | -0.076 (0.057) |
| **EPCAM** | -0.051 (0.133) | 0.052 (0.191) |
| **SOX10** | 0.235 (**<0.0001**) | **0.155 (<0.01)** |
| **SOX9** | -0.098 **(0.004)** | -0.012 (0.766) |

**Supplementary table 4a**: The association between BMI-1 expression and breast cancer stem cell markers (BCSC) at the protein level

**Supplementary table 4b**: The association between *BMI1* and breast cancer stem cell markers (BCSC) at the mRNA Level

| **BCSC markers** | ***BMI1* expression (Whole cohort)**  Correlation coefficient (p-value) | ***BMI1* expression (Luminal ER+BC)**  Correlation coefficient (p-value) |
| --- | --- | --- |
| ***ALDH1A1*** | -0.033 (0.138) | -0.058 **(0.025)** |
| ***ALDH1A3*** | -0.181 **(<0.0001)** | -0.058 **(0.026)** |
| ***CD133*** | -0.205 **(<0.0003)** | -0.054 **(0.038)** |
| ***CD44*** | -0.103 **(<0.0007)** | -0.115 **(0.0001)** |
| ***CD24*** | -0.232 **(0.002)** | -0.195 **(0.0004)** |
| ***EPCAM*** | 0.073 **(0.001)** | 0.123 **(0.0008)** |
| ***SOX10*** | -0.257 **(0.0001)** | -0.119 **(0.0006)** |
| ***SOX9*** | -0.011 (0.616) | 0.090 **(0.0007)** |
